# Proctors exploit three-dimensional ghost tools during clinical-like training scenarios: a preliminary study

**DOI:** 10.1007/s00345-016-1944-x

**Published:** 2016-09-26

**Authors:** Anthony M. Jarc, Andrew A. Stanley, Thomas Clifford, Inderbir S. Gill, Andrew J. Hung

**Affiliations:** 1 Intuitive Surgical, Inc., Norcross, GA USA; 2 Intuitive Surgical, Inc., Sunnyvale, CA USA; 3 Department of Urology, USC Institute of Urology, Keck School of Medicine, University of Southern California, Los Angeles, CA USA

**Keywords:** Telementoring, Proctor, Surgeon training, Ghost tools, Performance metrics, Augmented reality

## Abstract

**Purpose:**

In this study, we examine three-dimensional (3D) proctoring tools (i.e., semitransparent ghost tools overlaid on the surgeon’s field of view) on realistic surgical tasks. Additionally, we develop novel, quantitative measures of whether proctors exploit the additional capabilities offered by ghost tools.

**Methods:**

Seven proctor–trainee pairs completed realistic surgical tasks such as tissue dissection and suturing in a live porcine model using 3D ghost tools on the *da Vinci* Xi Surgical System. The usability and effectiveness of 3D ghost tools were evaluated using objective measures of proctor performance based on proctor hand movements and button presses, as well as post-study questionnaires.

**Results:**

Proctors exploited the capabilities of ghost tools, such as 3D hand movement (*p* < 0.001), wristedness (*p* < 0.001), finger pinch gestures (*p* < 0.001), and bimanual hand motions (*p* < 0.001). The median ghost tool excursion distances across proctors in the *x*-, *y*-, and *z*-directions were 57.6, 31.9, and 50.7, respectively. Proctors and trainees consistently evaluated the ghost tools as effective across multiple categories of mentoring. Trainees found ghost tools more helpful than proctors across all categories (*p* < 0.05).

**Conclusions:**

Proctors exploit the augmented capabilities of 3D ghost tools during clinical-like training scenarios. Additionally, both proctors and trainees evaluated ghost tools as effective mentoring tools, thereby confirming previous studies on simple, inanimate tasks. Based on this preliminary work, advanced mentoring technologies, such as 3D ghost tools, stand to improve current telementoring and training technologies in robot-assisted minimally invasive surgery.

**Electronic supplementary material:**

The online version of this article (doi:10.1007/s00345-016-1944-x) contains supplementary material, which is available to authorized users.

## Introduction

Surgical training has always required efficient mentorship, and this is not different for robot-assisted minimally invasive surgery (RAMIS) [1, 2]. The current standard is in-person instruction or demonstration by an attending surgeon (i.e., see one, do one, teach one). This approach stems from traditional open surgery where it is the most natural method of instruction. However, new technologies are being developed to facilitate and improve mentorship in-person and remotely [3–5]. In particular, RAMIS offers unique opportunities to facilitate and improve mentorship by placing a computer system between the surgeon and the patient [2].

If we take one particular RAMIS platform—the *da Vinci* Surgical System (Intuitive Surgical, Inc., Sunnyvale, CA, USA)—as an example, we see it includes technologies to support mentoring. Firstly, mentors can utilize two-dimensional (2D) telestration on the vision cart touch screen to draw directly over the operative field. Secondly, three-dimensional (3D), virtual pointers on an optional second console can be used by a proctor to point on a shared operative field [2, 6, 7]. Finally, *da Vinci* Connect™ enables remote proctoring from a laptop whereby the proctor can view the surgeon’s operative field and communicate through two-way audio and 2D telestration using her mouse [8].

Despite these technologies, there remains an opportunity to improve mentoring in RAMIS. In previous studies on both intra- and inter-hospital telementoring, we found that proctors frequently expressed a desire for 3D viewing and 3D telestration by the proctor [8, 9]. Thus, remote proctoring can be improved beyond 2D so that the trainee and proctor share a more similar view of the operative field. Similarly, the types of tools used to mentor in 3D can be improved from the current dual console “cone-shaped” pointers. 3D display and dynamic 3D mentoring tools would in theory permit more effective proctor demonstrations and more streamlined communication between the proctor and trainee. In turn, this could lead to improved surgeon training and patient safety.

We previously proposed 3D proctoring tools, in the form of semitransparent ghost tools overlaid on the surgeon’s field of view, to enable new and improved proctor–trainee interactions [10]. We demonstrated that 3D ghost tools were preferred by both proctors and trainees over conventional 2D tools during inanimate exercises. However, whether a subjective preference for ghost tools actually translates to effective use of these tools and their capabilities remains unclear. Furthermore, these evaluations were performed on inanimate training exercises which exhibit key differences from clinical tasks and scenarios where ghost tools may add additional value.

In the present study, we combined subjective evaluations of ghost tools with novel objective measures of whether proctors exploit the additional capabilities offered by ghost tools as an integrated 3D gesturing platform when mentoring realistic surgical tasks. We hypothesized that in a live surgical environment, proctors would utilize the features offered by ghost tools: 3D hand movements, hand orientation or wristedness, pinch gestures through ghost tool jaw open/close, and concurrent bimanual hand motions. Fundamental to this study, we utilized the intrinsic capability of ghost tools to measure proctors’ 3D hand movements and button presses to examine our hypotheses.

## Methods

### Proctoring setup

**Fig. 1:**
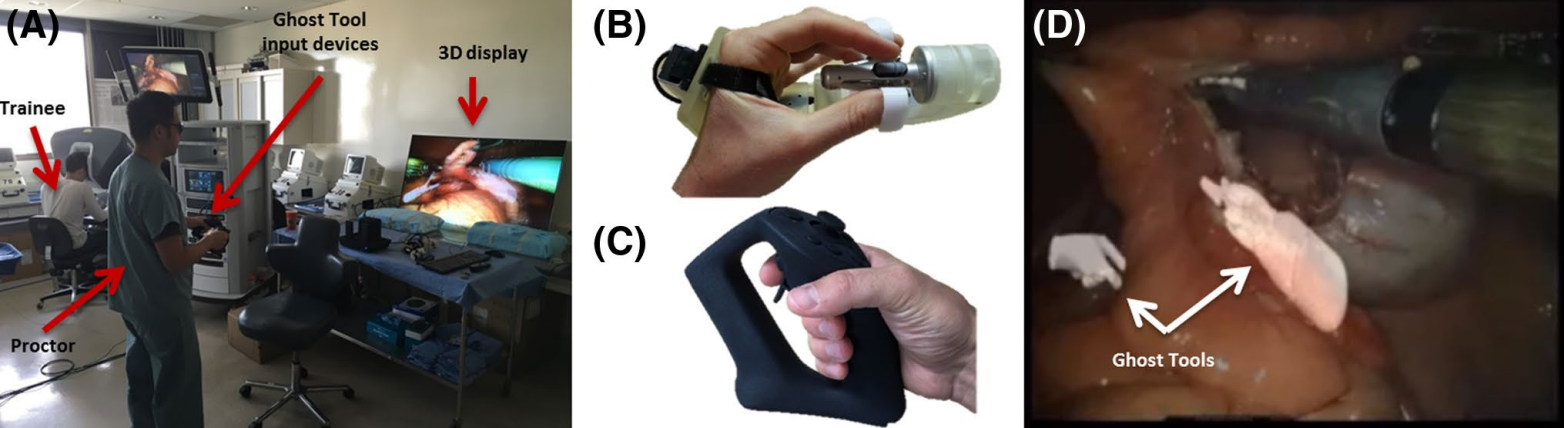
**a** Experimental setup with trainee, proctor, input devices, and three-dimensional display. **b** Custom wireless input device that resembles *da Vinci* hand controller at the surgeon console. **c** Commercial input device from Sixense, Inc. **d** Image of ghost tools being used for proctoring on a tissue task

The proctoring setup was nearly equivalent to our previous study (please refer to [10] and Fig. 1a). Briefly, we evaluated proctors’ use of 3D, semitransparent tools (called ghost tools) to mentor trainees during standardized clinical-like tasks on the *da Vinci* Xi™ Surgical System (Intuitive Surgical, Inc., Sunnyvale, CA, USA). Proctors and trainees were located in the same room although the platform can be made remote. The proctor used input devices (Fig. 1b, c) to control the position, orientation, and state of the ghost tools while viewing a 3D polarized display (EG9600, LG Corporation, Seoul, South Korea). Custom software was written to overlay the ghost tools on the stereoscopic endoscopic images streamed from the video outputs of the system (Fig. 1d). The resulting endoscopic images with ghost tool overlays were displayed to the trainee in a sub-window on the surgeon console using the 3D TilePro feature.

Distinct from our previous study, proctors used wireless input devices to control the ghost tools. The first input device was a custom wireless controller that used the same handle as the hand controllers on the *da Vinci* surgeon console and communicated data wirelessly through an XBee^®^ wireless module (Fig. 1b) (Digi International, Minnetonka, MN, USA). An alternate input device was an off-the-shelf game controller with its trigger and button states remapped to align with the capabilities of the *da Vinci* hand controllers (Fig. 1c) (Sixense Entertainment, Inc., Los Gatos, CA, USA). Proctors were able to freely choose between three different types of ghost tools—pointers, cartoon hands, and *da Vinci* instruments. The pointers and hands were able to draw line annotations in 3D. The virtual *da Vinci* instruments matched those used by the trainee for each task.

### User study

**Fig. 2:**
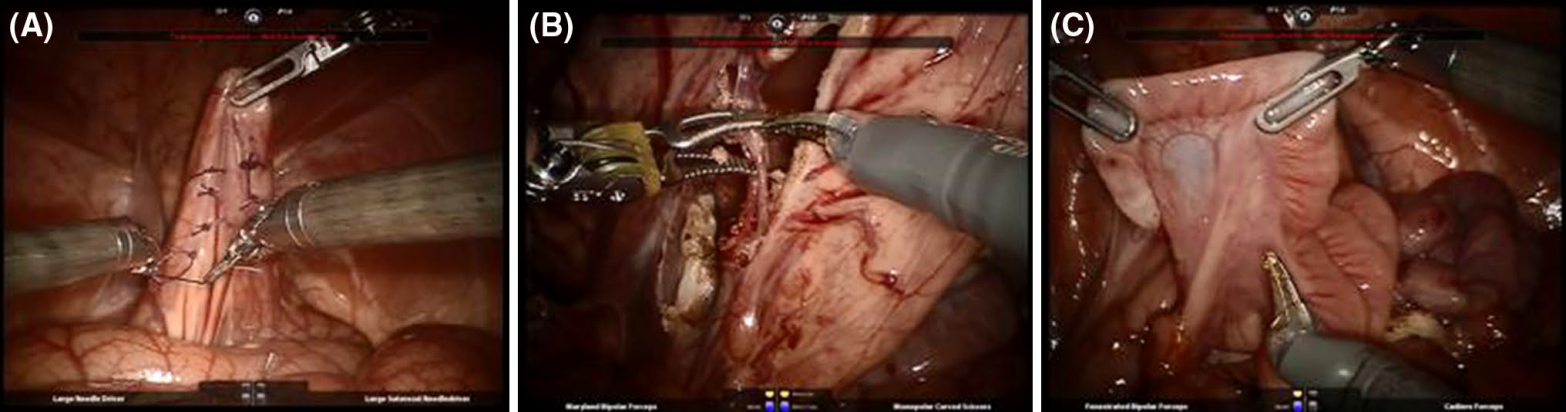
Three clinical-like training tasks on a live porcine model. **a** Task 1 (suturing): four interrupted sutures were thrown (two half hitches followed by a surgeon’s knot) on the surface of the sigmoid. Trainees used two large needle drivers with 3-0 vicryl (RB-1) cut to a length of 10 cm. Additional suture was supplied, if necessary. **b** Task 2 (dissection): the ureters were dissected by exposing a window between the medial and lateral leaves of the bladder suspensory ligament. A small grasping retractor was used in the fourth arm to retract the bladder anteriorly to place the suspensory ligaments under tension. Monopolar curved scissors and Maryland bipolar instruments were used for the dissection. **c** Task 3 (dissection): the left and right uterine horns were mobilized from the broad ligaments. Trainees worked medially starting at the fallopian tubes and ending at the uterine body. They readjusted their fourth arm to provide optimal retraction and used a combination of bipolar and monopolar energy. Finally, once both uterine horns were fully mobilized, trainees amputated the uterine body

The usefulness of ghost tools was examined through a user study at the Keck School of Medicine at the University of Southern California (Los Angeles, CA, USA). This study addressed a limitation of our previous work [10]—the simplicity of the training tasks in an inanimate environment. Here, we studied ghost tools during three clinical-like tasks on a live porcine model (female pigs, 38–40 kg) to closely resemble actual steps of a clinical procedure (Fig. 2, see caption for task descriptions). The technical skills varied across the exercises and included EndoWrist^®^ manipulation, suture management, knot tying technique, energy application, retraction, camera movement, and fourth arm use. The training tasks were broadly categorized as suturing (task 1) or dissection (tasks 2 and 3) for group analysis (see below).

Proctors and trainees were randomly paired together to complete the three training exercises on the porcine model. Proctors consisted of experienced RAS surgeons (median 375, range 225–4500 cases). Trainees were medical students or surgical residents (PGY2–PGY6). Participation followed an institutional animal care and used committee-approved protocol. Each proctor–trainee pair received standardized instructions on how to complete the exercises from a consistent, experienced RAS trainer (>100 surgeons trained) immediately prior to the given exercise. Then, proctors were given additional standardized instructions on how to use the ghost tools with both input devices. They spent two minutes familiarizing themselves with the controls and capabilities. Proctors were instructed to provide mentoring to the trainee on technique, errors, or inefficiencies as they saw fit. They could choose whichever ghost tool they preferred and could switch at any point during an exercise. The order of the training exercises and the input device used by the proctor were randomly assigned.

### Measures

**Fig. 3:**
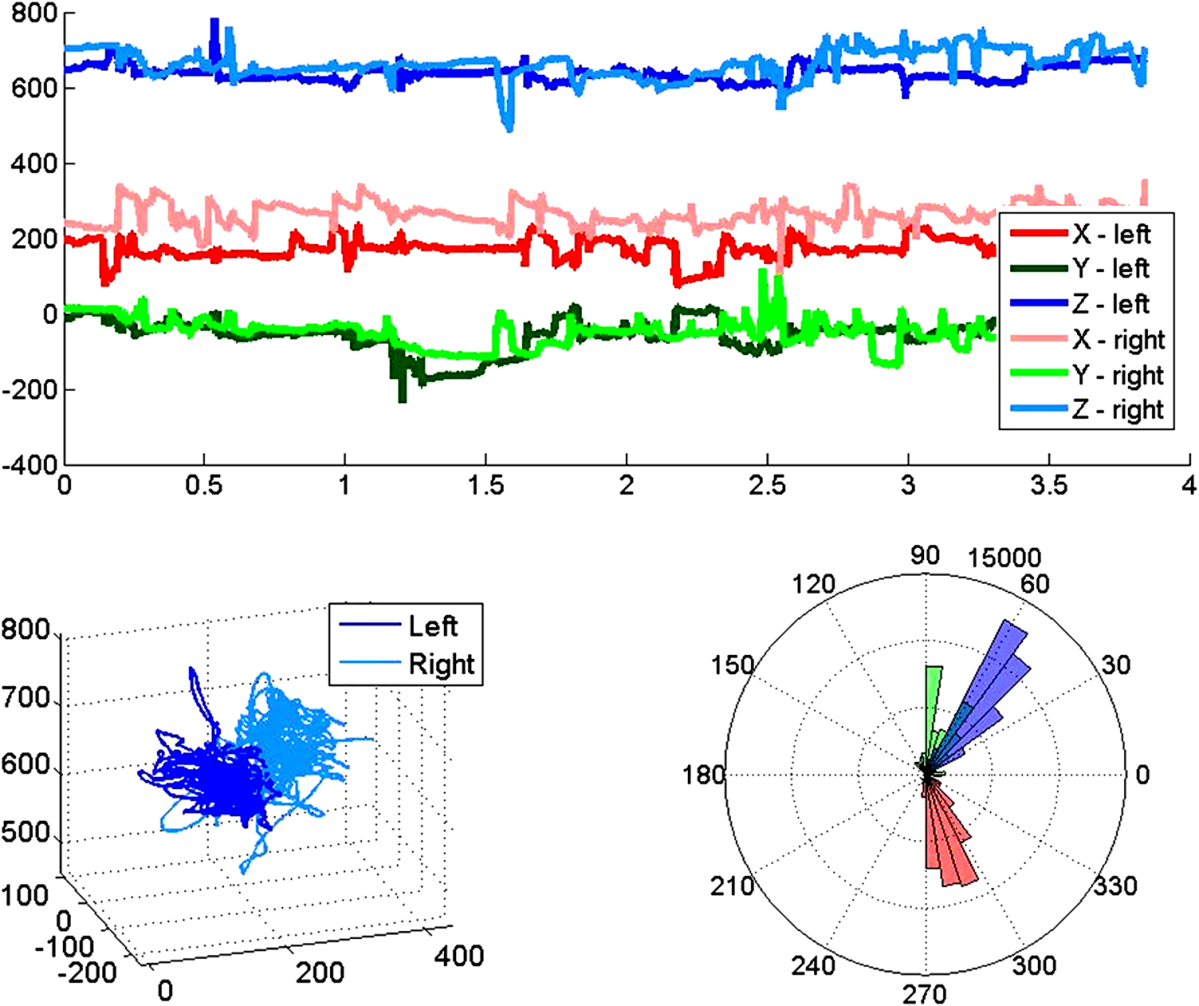
Data of proctor hand movements (right hand/left hand) (**a** time series, **b** three-dimensional trajectories) and hand orientation (**c** roll (*red*), pitch (*green*), and yaw (*blue*) angles)

Video (30 Hz) and audio were recorded during each training exercise for all proctor–trainee pairs. In addition, the proctors’ hand movements and button presses were recorded from the ghost tool devices at 50 Hz and synchronized to the video recordings. These measures included the six degrees of freedom that represented pose (i.e., 3D position and orientation), the trigger state (i.e., opening and closing of the ghost instrument jaws), master clutch events (i.e., when the proctor chooses to make ergonomic adjustments by moving his or her hands without the ghost instruments following), and home events (i.e., an efficient way to reset the virtual instruments to a neutral position within the operative field) of each proctor input device. For both input devices, the pose was tracked using the electromagnetic tracker in the Sixense system. For the custom wireless controller (Fig. 1b), the trigger state and events were recorded using custom software that logged the XBee wireless communication. For the off-the-shelf game controller (Fig. 1c), the trigger state and events were recorded using the Sixense application program interface (API). Example Cartesian trajectories of a proctor’s hands are shown in Fig. 3a, b. Figure 3a shows the *x*-, *y*-, and *z*-movements of the proctor’s hands over a designated time window and closely mirrors the type of data that can be recorded from the operating surgeon at the surgeon console. Figure 3b shows the proctor’s hand movements in 3D, illustrating the proctor’s hands remained in a compact workspace with few large excursions. Example orientations of a proctor’s wrists are shown in Fig. 3c. Hand roll (red), pitch (green), yaw (blue) angles deviated from a neutral orientation sparingly, possibly during brief, pointed demonstrations. To the authors’ knowledge, this is the first report of direct, quantitative measurement of proctor hand movements in 3D.

After completing all three training exercises, both proctors and trainees completed GEARS evaluations [11] and post-questionnaires. The post-questionnaire was the same, validated questionnaire as used in previous studies [10, 12] and evaluated the proctoring tools across six categories on a five-point scale. Note that a single subjective survey encompassed all three training exercises and all ghost tool interactions for each proctor–trainee pair.

Finally, an expert observer (AJH) evaluated all proctors in terms of their use of ghost tools using an observer questionnaire. Seven questions were on a five-point scale and evaluated how effectively proctors provided mentoring (see Table 2). The remaining two questions estimated the proportion of time during each mentored session that proctors spent utilizing ghost tools and verbal guidance.

### Analysis

The quantitative measures of proctor behavior as well as the survey responses were analyzed. The quantitative measures were used to examine whether proctors actually exploited certain features of ghost tools that differentiate them from currently available proctoring tools. Prior to extracting any features, the data were filtered to exclude periods of inactivity (e.g., no movement by the proctor) and any irrelevant movements (e.g., picking up/setting down the ghost tools or spurious movements as defined below). Firstly, the position data of the ghost tools were smoothed with a Gaussian filter (window size = 20 frames). Next, the Euclidean distance the ghost tool traveled for each time step was computed. The *x*-direction corresponded to left/right movements, the *y*-direction corresponded to up/down movements, and the *z*-direction corresponded to in/out movements all with respect to the endoscopic field of view. Inactivity was defined as any instances with a change in distance less than a lower threshold (0.1 cm/frame). Similarly, irrelevant movements were defined as any instances with a change in distance greater than an upper threshold (5 cm/frame) or an actual distance too far from the mean position of the proctor hand position (200 cm). These thresholds were determined empirically by examining the raw data across subjects. Inactivity and irrelevant movements were removed from all data despite being derived solely from position data.

Once the data were filtered, features that quantified 3D movements (3DMOVE), wristedness (WRIST), trigger use (TRIG), and bimanual instruction (BIMAN) were computed. 3DMOVE and WRIST were decomposed into individual dimensions (*x-*, *y-*, and *z*-movements and roll, pitch, and yaw orientations, respectively). The interquartile range (IQR) was used to calculate the variability of these features. For 3DMOVE, the interquartile range was computed for the filtered position data. Similarly for WRIST, the interquartile range was computed for the filtered orientation data.

TRIG usage corresponded to the open/close action of the hand fingers or instrument jaws. Open/close actions were used by proctors to indicate trainees how to grasp tissue or suture, how to perform blunt dissection, and how to position her instruments in 3D space. TRIG was quantified as the number of times (count) the trigger exceeded 20 % of the closed position for both of the ghost instruments.

Finally, BIMAN measured when proctors used both hands simultaneously to provide instruction rather than just one hand. Bimanual dexterity is often used in surgeon skill assessment, such as question two in GEARS [11], but is typically derived from expert video review rather than directly from device data streams. BIMAN was quantified as the ratio of time when both hands were used to the total task time.

Student’s *t* tests with a significance level of *p* < 0.05 were used to compare the quantitative features to zero (i.e., if feature was used at all), across different components (e.g., *x*-movement vs *z*-movement), across task types (e.g., dissection vs suturing), and across handedness (i.e., dominant vs non-dominant hand).

The median and range of responses to the survey questions were reported across trainees and proctors, separately. Two categories were created for each question that corresponded to “agree”/“optimal” and “disagree”/“sub-optimal,” depending on the particular question. All GEARS questions and most post-questionnaire and observer questionnaires were on a five-point scale where 1 corresponded to “disagree” and 5 corresponded to “agree.” In this way, positive responses were 4 or 5 and negative responses were 1, 2, or 3. For several questions for the observer questionnaire, 3 was the best response. In these cases, the “optimal” category consisted of a response of 3 and the “suboptimal” response consisted of all other responses (see Table 2). Chi-square tests were used for statistical comparisons with *p* < 0.05. Significance tests compared the survey data to an expected response of 50 % chance in each category. Overall comparisons for all GEARS and post-questionnaire responses between proctors and trainees were performed using a Mann–Whitney *U* test with *p* < 0.05.

## Results

Seven trainees and six proctors participated in the research study. Two trainees were medical students, one was PGY2, one was PGY3, one was PGY4, and two were PGY6. Three trainees were new (0 cases) to the *da Vinci* Surgical System, three were intermediate (1–99 cases), and one was expert (>99 cases). This group of trainees enabled us to provide a preliminary characterization of relevant proctor–trainee interactions across a wide range of trainee skill levels—something proctors encounter routinely. The six proctors were very experienced on the *da Vinci* system (median 375, range 225–4500 cases). One proctor participated in the study twice but with different trainees. Five proctors were in urology, and the remaining one was in gynecology. Informed consent was obtained from all individual participants included in the study.

Each proctor used both the custom controller and the game controller for at least one task as part of the study. In total, the custom controller was used for twelve training tasks and the off-the-shelf game controller was used for nine training tasks.

### Proctor tool data

**Fig. 4:**
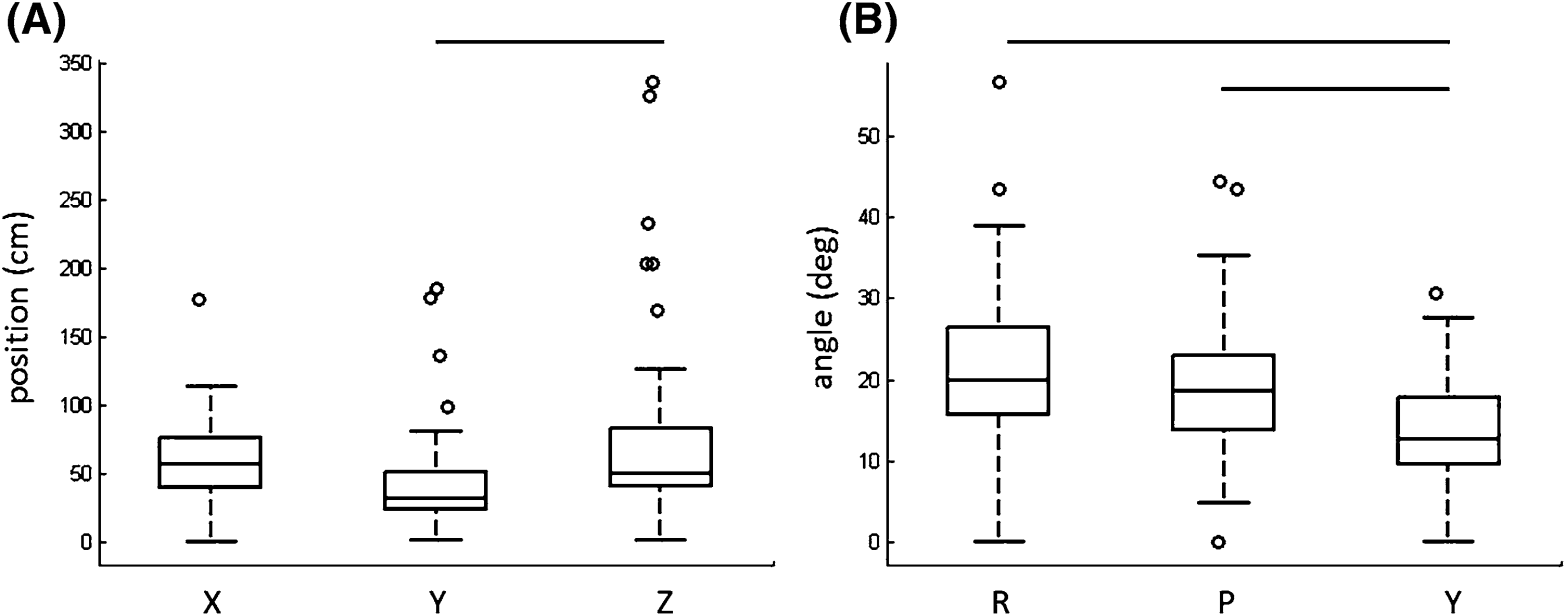
**a** 3D movement interquartile ranges for *x-*, *y-*, *z*-movement directions. **b** Wrist angle interquartile ranges for roll (*R*), pitch (*P*), and yaw (*Y*) angles. Right and left hand data combined. *Horizontal black lines* indicate significant differences (*p* < 0.05) between groups

Proctors consistently moved in 3D (the 3DMOVE feature differed significantly from zero (*p* < 0.001)). The median excursion distances (centimeters) across proctors in the *x*-direction, *y*-direction, and *z*-direction were 57.6, 31.9, and 50.7, respectively. The corresponding interquartile ranges for the *x*-direction, *y*-direction, and *z*-direction were 40.6–77.0, 23.9–51.7, and 40.9–84.0, respectively. Excursions in the *z*-direction were significantly greater than excursions in the *y*-direction (*p* < 0.05; Fig. 4a). If proctors moved in two dimensions, which closely parallels the type of instruction from telestration on the touch screen or through da Vinci Connect [8], the *z*-direction excursions would not differ significantly from zero (or the *x*- and *y*-directions). Therefore, when given the ability to provide instruction to trainees in the same rich 3D environment, proctors consistently utilized 3D movements.

Similar to 3D movements, proctors consistently manipulated the orientation of the ghost tools: The WRIST feature significantly differed from zero (*p* < 0.001). The median angular excursions (degrees) across proctors in roll, pitch, and yaw were 20.0, 18.6, and 12.6, respectively. The corresponding interquartile ranges for roll, pitch, and yaw excursions were 15.6–26.4, 13.8–23.0, and 9.6–17.9, respectively. Roll and pitch excursions were significantly greater than yaw excursions (*p* < 0.01; Fig. 4b). Proctor instruction on instrument orientation seems helpful to optimize trainee performance given the wristed capabilities of RAMIS instrumentation (which differs from conventional laparoscopic instruments which lack wrists).

Proctors also used the ability to open and close the ghost tool jaws (TRIG) (*p* < 0.001) and both hands simultaneously (BIMAN) (*p* < 0.001). These statistical tests compared usage of TRIG and BIMAN to no usage. The median count of TRIG events was 21 (range 9–56.5). The median amount of time spent providing instruction with both hands simultaneously was 30.5 % (range 25.1–39.0 %). These additional features—open/close gestures and bimanual instruction—suggest value in proctor tools that exhibit similar features to actual instruments on RAMIS systems.

Both non-dominant and dominant hands were used across all features (*p* < 0.001). However, no feature usage differences existed between dominant and non-dominant hands for any of the features. Similarly, both suturing and dissection tasks used features similarly (*p* < 0.001). Again, however, suturing and dissection tasks were not significantly different across all features. Finally, hand dominance did not impact proctor tool use within or across task types.

Additional events (or button presses), such as master clutch, were utilized but not frequently or consistently enough across proctors to reach significance (*p* > 0.05).

### User questionnaires

Proctors and trainees evaluated all four types of proctoring tools favorably (median responses were ≥4 (out of 5) across all categories from the post-questionnaire; see Table 1). Proctors responses indicated a significant difference in three of eight questions. They included: Ghost tools helped them recognize anatomy, enabled the trainee to operate safely, and worked smoothly (*p* < 0.05). Trainee responses reached significance (*p* < 0.05) on five of eight questions, including improving surgical/technical skills, improving confidence, allowing safe completion of task, and working smoothly. When comparing responses across all questions, trainees evaluated ghost tools more favorably than proctors [*p* < 0.05, trainee median 5 (range 2–5), proctor median 4 (range 3–5)]. There was no significant difference between proctor and trainee (self) GEARS evaluations.

**Table 1:** Trainee and proctor responses to questionnaire

Prompt	Trainee	Proctor
T: The proctoring helped me recognize ANATOMICAL structures P: The proctoring helped me delineate ANATOMICAL structures	5 (3–5)	**4 (4**–**5**)
T: The proctoring helped me improve my SURGICAL/TECHNICAL skills P: The proctoring helped the trainee improve their SURGICAL/TECHNICAL skills	**5 (4**–**5)**	4 (3–4)
T: The proctoring improved my CONFIDENCE as a surgeon P: I was CONFIDENT in my ability to proctor	**4 (4**–**5)**	4 (3–4)
T/P: The proctoring allowed for SAFE completion of the task	**5 (3**–**5)**	**4 (3**–**5)**
T/P: The proctoring experience WORKED smoothly	**5 (3**–**5)**	**4 (3**–**5)**
T/P: The proctoring interface was EASY TO USE	**4 (2**–**5)**	4 (3–5)
T/P: The 3D feature was helpful	4 (2–5)	4 (3–5)
T/P: The 3D feature was more helpful than 2D	4.5 (2–5)	4 (3–5)

Responses were on a five-point scale. Values are reported as median with range in parentheses

*T* trainee prompt, *P* proctor prompt

Bold denotes significant difference between “agree” versus “disagree” responses (*p* < 0.05, Chi-square test)

The expert reviewer evaluated proctors favorably across all questions except one (see Table 2). The question that was slightly less than favorable indicated that the expert observer believed the ghost tools were underutilized by the proctor across the training exercises. The expert observer’s responses reached significance for five of seven questions, including positive evaluation of the overall intervention and the effectiveness, frequency, and balance of proctors’ interventions (*p* < 0.05). Finally, the proctors used verbal instruction significantly more than ghost tool instruction [*p* < 0.01; verbal 60 % (55–75 %) and ghost tool instruction 40 % (25–45 %)].

**Table 2:** Expert observer responses to questionnaire

Prompt	Observer	“Agree” responses	“Disagree” responses
The proctor intervened and provided mentoring when needed.	5 (4–5)	4, 5	1, 2, 3
The proctor’s intervention was successful.	**5 (4**–**5)**	4, 5	1, 2, 3
How effectively did the proctor intervene?	**4 (3**–**5)**	4, 5	1, 2, 3
How frequently did the proctor intervene?	**3 (3**–**4)**	3	1, 2, 4, 5
How balanced was the proctor’s use of verbal direction and mentoring tool?	**3 (2**–**3)**	3	1, 2, 4, 5
Proctor’s verbal direction was…	**3 (3**–**4)**	3	1, 2, 4, 5
Proctor’s mentoring tool was…	2 (2–3)	3	1, 2, 4, 5
Verbal %	60 (55–75)		
Tool %	40 (25–45)		

Responses were on a five-point scale. Values are reported as median with range in parentheses

Bold denotes significant difference between “agree” versus “disagree” responses (*p* < 0.05, Chi-square test)

There were no significant correlations between GEARS, post-questionnaire, and quantitative proctor action measures (*p* > 0.05).

## Discussion

In this research, we provide preliminary evidence that proctors utilize the features of 3D ghost tools during porcine training exercises. In particular, we used a novel data stream of proctor hand movements and button presses in combination with survey data to rigorously quantify how these features were indeed exploited by proctors. This work extends our previous research [10] by: (1) examining ghost tools on live tissue training exercises as opposed to dry-laboratory models that simply lacked many aspects of clinical surgery, such as realistic tissue properties, energy application, and anatomical landmarks, and (2) using proctor input devices that more closely resembled the actual surgeon console. It has continued to move us closer to our long-term goal of outfitting proctors with the optimal tools to provide effective instruction to trainees.

Our approach to characterize how proctors use novel input devices to provide instructions to trainees parallels how virtual reality simulators measure surgeon hand and instrument movements and system events to evaluate performance using metrics, such as economy of motion, master workspace range, and energy application. However, different from these studies, we evaluate the hand movements and button presses of proctors who are not controlling the actual system but instead who use ghost tools as instructional aids. Figure 4 and the additional measures in Results section show proctors exploit the capabilities of advanced proctor tools consistently by moving in 3D (3DMOVE), specifying exact ghost tool wrist orientations (WRIST), demonstrating grasping actions (TRIG), and demonstrating bimanual dexterity (BIMAN). Clear evidence that proctors exploit these capabilities is essential to further optimize how proctors and trainees interact.

We envision rich proctoring interactions like those enabled by ghost tools to complement other training activities as surgeons move through their learning curves [13–15]. These include in-person and remote case observation, in-person proctoring, and focused training on virtual reality simulators, dry-laboratory models, porcine tasks, and cadavers. Current technologies facilitate instruction but offer much room for improvement. For example, two-dimensional (2D) telestration can be used by proctors either in-person on a touch screen or remote through solutions like *da Vinci* Connect. Ghost tools offer improvements over 2D telestration through richer 3D interactions, a 3D view for the proctor, and a relatively low-cost, mobile platform that can be flexibly used in-person or remotely.

Relatedly, a dual console setup delivers a 3D view to proctors as well as 3D pointers while also allowing a proctor to assume control of the surgical instruments, possibly to demonstrate a particular skill [6, 16]. Although several features of ghost tools overlap with features of a dual console, there remain critical use cases for both technologies. Dual consoles remain essential when exchange of instrument control is needed without either the proctor or trainee stepping away from the surgeon console, whereas ghost tools do not control the system and require proctors illustrate technique simply using semitransparent overlays. If actual control is unneeded or infrequent, ghost tools offer a low-cost, mobile alternative. Furthermore, ghost tools extend the features of dual console 3D pointers to appear as ghost hands or *da Vinci* instruments, to telestrate in 3D, and to animate the grasping behavior of instruments [10]. Additionally, as mentioned above, we rigorously evaluate these features by studying quantitative metrics of proctor behaviors, which is a level more detailed than previous studies on dual console use [6].

There exist many potential applications to exploit the novel data streams from 3D proctor input devices. Firstly, it could be used to provide performance feedback to proctors similar to virtual reality simulators to improve how proctors interact with trainees. Secondly, one could characterize different proctor styles to optimally pair proctor–trainee pairs. Finally, these data could be used to systematically improve the designs of proctor tool input devices and features based on ongoing use [17].

Limitations exist with this study. Firstly, although we studied proctor–trainee interactions on clinical-like tasks, they still differed from actual clinical scenarios. In clinical situations, surgeons must deal with the stress, anatomical variation, and critical decision making associated with operating on actual patients, which remains appreciably different than operating on a porcine model. A second limitation was that our quantitative measures evaluated the usability of proctor tools but did not include analysis of trainee performance as a result of proctor interventions or contextual information. One could mine operative videos or utilize *da Vinci* system data from trainee movements to begin to gather such insights. Yet another limitation was the number and demographics of subjects in this study, particularly trainees whom encompassed a wide range of skills. A larger subject population across several different specialties (i.e., urology, gynecology, general surgery, etc.) is needed to develop a more thorough understanding of proctoring interactions. Nonetheless, the authors strongly believe the preliminary data presented in this study help justify further investigation and development in this technology. Finally, proctors and trainees were in the same room for this study. It will be essential to examine truly remote proctoring scenarios using ghost tools.

## Conclusion

In this study, we show that proctors exploit the augmented capabilities of 3D ghost tools through novel quantitative measures. Furthermore, proctors and trainees both favorably evaluate ghost tools as an improvement on current technologies. In the future, we believe novel objective measures, similar to those reported here, could be used to more precisely measure proctor performance and proctor–trainee interactions, as well as to develop guided training scenarios for proctors similar to virtual reality training exercises for console surgeons. Furthermore, we anticipate advanced mentoring technologies, such as ghost tools, to be valuable for remote training, credentialing, and telementoring, especially as RAMIS expands globally [18, 19].

## Electronic supplementary material

Below is the link to the electronic supplementary material.

Supplementary material 1 (MP4 17962 kb)
